# Routine activities and emotion in the life of dairy cows: Integrating body language into an affective state framework

**DOI:** 10.1371/journal.pone.0195674

**Published:** 2018-05-02

**Authors:** Daiana de Oliveira, Linda J. Keeling

**Affiliations:** Department of Animal Environment and Health, Swedish University of Agricultural Sciences, Uppsala, Sweden; Boston Children’s Hospital / Harvard Medical School, UNITED STATES

## Abstract

We assessed dairy cows’ body postures while they were performing different stationary activities in a loose housing system and then used the variation within and between individuals to identify potential connections between specific postures and the valence and arousal dimensions of emotion. We observed 72 individuals within a single milking herd focusing on their ear, neck and tail positions while they were: feeding from individual roughage bins, being brushed by a mechanical rotating brush and queuing to enter a single automatic milking system. Cows showed different ear, neck and tail postures depending on the situation. When combined, their body posture during feeding was ears back up and neck down, with tail wags directed towards the body, during queuing their ears were mainly axial and forward, their neck below the horizontal and the tail hanging stationary, and during brushing their ears were backwards and asymmetric, the neck horizontal and the tail wagging vigorously. We then placed these findings about cow body posture during routine activities into an arousal/valence framework used in animal emotion research (dimensional model of core affect). In this way we generate *a priori* predictions of how the positions of the ears, neck and tail of cows may change in other situations, previously demonstrated to vary in valence and arousal. We propose that this new methodology, with its different steps of integration, could contribute to the identification and validation of behavioural (postural) indicators of how positively or negatively cows experience other activities, or situations, and how calm or aroused they are. Although developed here on dairy cattle, by focusing on relevant postures, this approach could be easily adapted to other species.

## Introduction

It is well known that behaviour is influenced by the emotional state of the animal and in recent years there has been an increasing interest in how changes in body posture might be an external indicator of this internal state. There are many definitions of emotion (e.g. [1] [2]) but here we use the term in a similar way to de Vere and Kruczaj [3], as an all-encompassing concept to cover feelings, affect and mood. Generally speaking, an animal moves towards things or repeats activities it experiences as positive and avoids things it experiences as negative [4, 5]. In this way emotion can be considered adaptive [4, 6]. Indeed already Darwin illustrated different body postures and facial expressions that were associated with fear and aggression [7] and in cattle these two emotional states have been shown to be linked to production and health [8, 9]. But it is very likely that cows, like other mammals including humans, experience a dynamic and varied emotional daily life.

Research has developed in the field of body postures of farm animals as indicators of their emotions (e.g. sheep [10–12], goats [13], pigs [14], cattle [15] [16]) in part because of the importance of emotion for animal welfare [17]. These studies have focused on specific body parts and so far, there is considerable variation among species and, in some cases, the same ear or tail postures are associated with opposing emotional states. This suggests that firstly, body postures and expression of emotion may not be generalized among species and, secondly, that specific postures may not be specific to single emotional states or have a different meaning when assessed alone or when combined to a composite, whole body posture of an individual animal. Moreover, there is a lack of research systematically assessing the body postures of animals in their normal living conditions. A better understanding of the variation in body postures in undisturbed animals might have practical implications for the development of on-farm welfare assessment e.g.[18] [19]. Even if there are technological advances (see [20] for a recent overview) the most frequently used method that is currently based on the reading of a whole animal´s body posture is “qualitative behaviour assessment” (QBA) [21]. This assessment is based on the human’s interpretation of how animals are feeling. It consists of 20 or so descriptors i.e. active, happy, fearful, etc. rated on a scale, and while useful in some situations, it has been criticised because of expectation bias by observers [22] and unsolved issues related to the inter and intra-observer reliability [23].

The aim of our study was to identify variation in the body posture of dairy cows focusing on ear, neck and tail positions while they are engaged in different routine activities. Furthermore, we aimed to use this variation to make predictions linking specific body postures, or combinations of postures with where the animal might be located in the two dimensional model of core affect [24] [25]. In this model, rather than attributing discrete emotions to an animal, its emotional state is described in terms of its valence (positivity and negativity) and its arousal [26]. We see this generation of predictions to be tested in future studies as a necessary first step in order to examine the emotional content of these activities, while avoiding the risks associated with observing animals in situations already thought to induce particular emotions. We selected the activities; brushing, feeding and queuing to be milked, because they are relatively frequent stationary activities during which we could reliably assess differences in posture. The wider aim is a methodology that can be applied irrespective of species.

## Material and methods

### Animals and housing

Our study was carried out at the Swedish Livestock Research Centre, Lövsta, Uppsala, Sweden. The type of housing and the facilities within it, as well as the total number, breed and age of the dairy cows were therefore those available during the experimental period. The studied herd was a mixed group of Swedish Red (68%) and Holstein (32%) dairy cows. The average group size was 55.46 ± 0.25 (mean±se) and data comprised 72 cows with an average lactation number of 2.16 ± 1.52 and average milk yield of 30.9 ± 0.6kg/day.

The cows were in a loose housing system with an automatic milking system (De Laval VMS^™^). They had access to two mechanical rotating brushes (DeLaval swinging cow brush SCB), 10 individual roughage feed bins (biocontrol CRFI), two concentrate feeders, a waiting area before the automatic milking system (AMS) and a resting area consisting of 24 cubicles with rubber mattresses scattered with wood shavings. The farm used a guided cow traffic system with passage gates to steer cows towards the feeding, milking or resting areas. All the cows in the study were managed according to the standard routine procedures of the farm. The study was approved by the Ethical Committee of Animal Experimentation in Uppsala, Sweden, under protocol C58/13.

### Behavioural observations

Observations began in September 2013 and were carried out during 20 days, from 9:00 to 17:00h within a period of 9 weeks. During the observation period cows were individually identified with a large number on their back using non-toxic spray and remarked weekly. Six trained observers worked in pairs. On each day one observer was responsible for brush 1 and the queuing area to the AMS (distance to cows >3m), while the other observer was responsible for brush 2 and the roughage feeding bins area (distance to cows > 10m). In the afternoon, they swapped responsibilities. The cows on this university farm were already used to different people, but they had additional time to habituate to the presence of the observers in this study when the ethogram was being developed and checked for inter-observer reliability.

The direct behaviour observations were gathered each day, during 12 observation periods evenly distributed over the day. Each observation period lasted 10 min and consisted of a series of instantaneous scans performed on the animals engaged in brushing, eating roughage or queuing to be milked. Starting with one of these cow, the first beep would signal the time to look at the position of the ears, 3 sec later at the second beep the position of the neck of the same cow was noted and at the third beep the position of the tail. The observer then moved to the next cow, and so on, during the 10-min period, so observing the identity and body positions of all cows performing the activities of interest. Cows could be observed more than once in each observation period, new cows could be included if they started to brush, feed or entered the queuing area, whereas observations on others cows ended if they stopped brushing, feeding or left the queue to enter the milking machine.

The activities were defined as follows:

1. **Brushing**: starts when the cow first touches the brush with any part of its body until it is no longer touching it, or the brush stops rotating despite the cow still being in contact with it. It is considered a new visit if the brush stopped rotating for at least 10 sec or when there was no contact for at least 10 sec.
2. **Feeding**: when the cow is located at the roughage bin with its head through the feeding rack. Both ears must be on the roughage bin side of the barrier and there should be food in its mouth or the cow is chewing. If the activity is interrupted for more than 30 sec, then the feeding bout is stopped.
3. **Queuing**: when the head and a front leg of the cow have crossed the entrance gate of the waiting area to the AMS and ends when the cow enters the robot to be milked.

**Fig 1:**
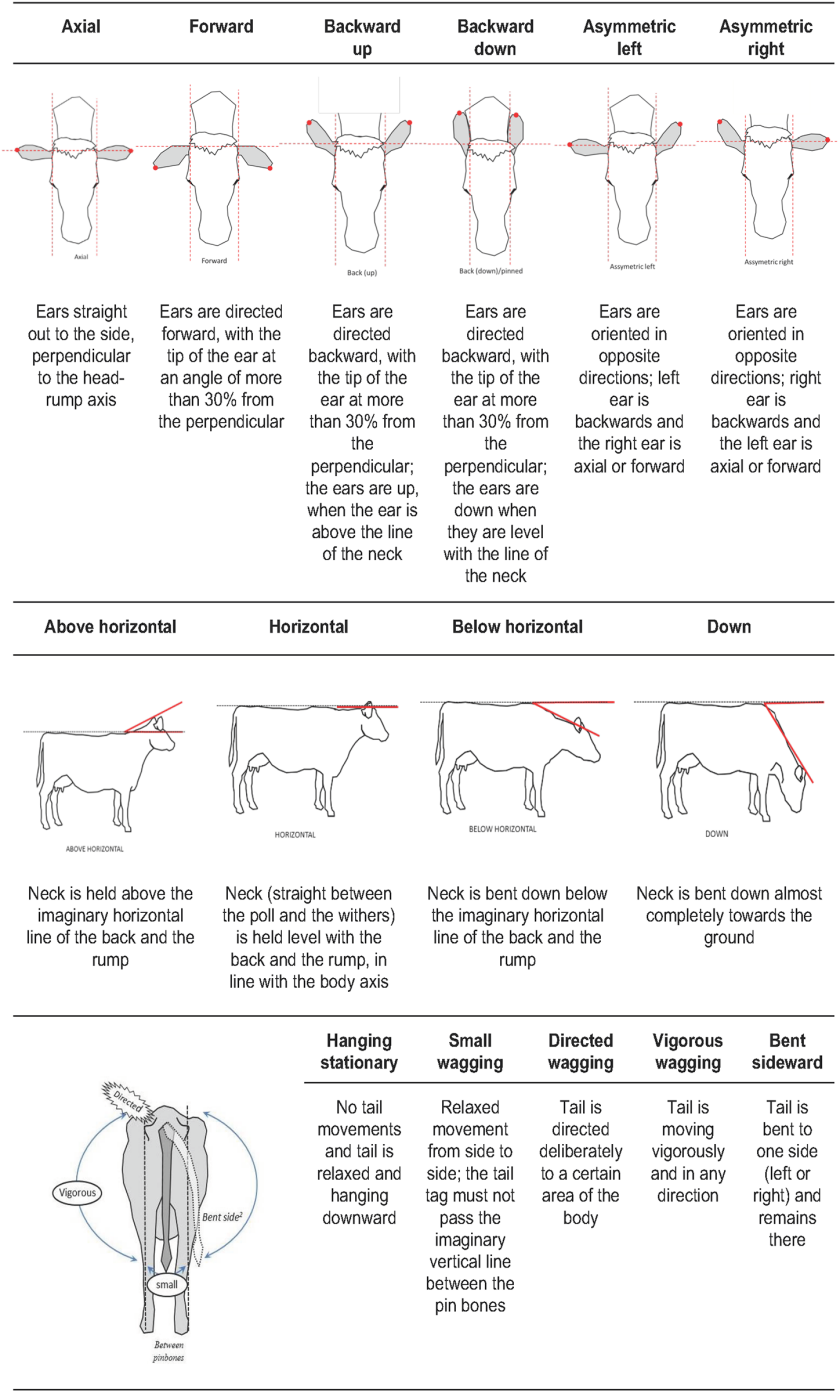
Diagrammatic representation of cows’ ear postures (axial, forward, backward up, backward down, asymmetric left, asymmetric right), neck postures (above horizontal, horizontal, below horizontal, down) and tail postures (hanging stationary, small wagging, directed wagging, vigorous wagging, bent sideward) observed while cows were brushing, feeding and queuing to be milked.

The ethogram to record ear, neck and tail positions is presented in Fig 1.

### Statistical analyses

The frequency of the different ear, tail and neck positions shown in each activity (brushing, feeding and queuing) was analysed using a generalized linear mixed model (PROC GLIMMIX in SAS 9.3, SAS Institute, Cary, NC, U.S.A.), with a binomial distribution. In the models, the behavioural postures (the 6 ear positions, 4 neck positions and the 5 tail positions) were included as response variables. Breed, week, period of the day, the different activities (brushing, feeding and queuing) and their interactions were included as fixed factors. Individual cow was considered as random effect. After testing the full model, the non-significant interactions and main effects were dropped sequentially, in a backwards elimination model selection. This model simplification method proceeded until we obtained a minimum adequate GLMM that included only terms significant at the *P* < 0.05 level. The final models were confirmed using a forward procedure with the Akaike information criterion (AICC). A Tukey post-hoc test was used for the comparisons.

To understand how these different specific ear, neck and tail positions contributed to the overall body posture, a principal component analysis (PCA; Joliffe 2002) was carried out with the data from all three activities using a correlation matrix. The Kaiser criterion was used for the number of retained factors (eigenvalue >1) and, after extraction, factors were subjected to a VARIMAX rotation. Loadings higher than (+/-) 0.4 were considered for interpretation. The PCA was then inspected and concentration ellipses were plotted as an indication of how the clusters of each activity differed from each other. These analyses were carried out in R software (R Core team 2013). The results are presented as mean ± standard errors.

## Results

The data were analysed from 961 observations of brushing, 1366 observations of feeding and 1437 observations of queuing to be milked. The average number of observations per cow (mean ± standard error) was 14.34 ± 1.36 for brushing, 20.39 ± 1.52 for feeding and 21.45 ± 1.54 for queuing to be milked.

We found significant differences between the positions of the ears, neck and tail of the cows during the different activities and, in the combined analysis, these were clustered making it possible to describe the typical body posture of a cow performing the routine activities of brushing, feeding or queuing to be milked in the stable. No significant effects were found of breed, week and period of the day.

### Ear positions

**Fig 2:**
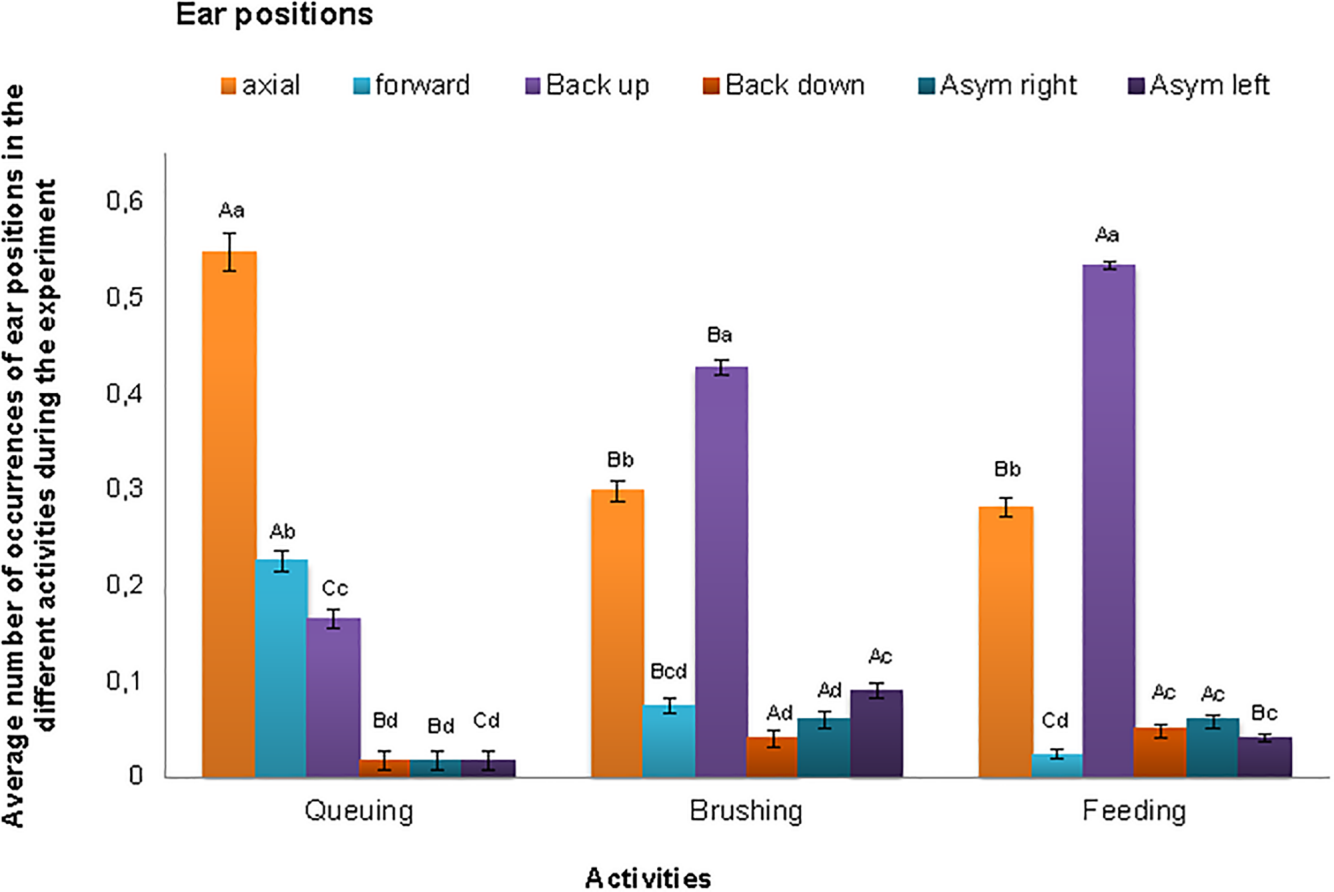
Average number of occurrences (±SE) of axial, forward, backward (up and down) and asymmetric (right and left) ears positions by dairy cows while queuing, brushing and feeding in the loose housing stable. Different lower-case letters (a, b, c, d) means that there is a difference within activities whereas different upper-case letters (A, B, C) means a difference between activities. Significant differences are presented at the 0.05 level.

There was a significant interaction (F = 48.6, df = 10, df error = 16431, P<0.001) between ear position and activity (Fig 2). An axial orientation was the predominant ear position during queuing, while for brushing and feeding the back-up ear position was the most frequently observed. The forward ear position decreased in frequency from queuing to brushing, being least frequent when the cow was feeding. Having only the right ear back (an asymmetric right ear position, Fig 1) was more common during brushing and feeding than during queuing. Having only the left ear back (an asymmetric left ear position) was most frequently observed while the cow was brushing, less so when it was feeding and least frequently when queuing.

### Neck positions

**Fig 3:**
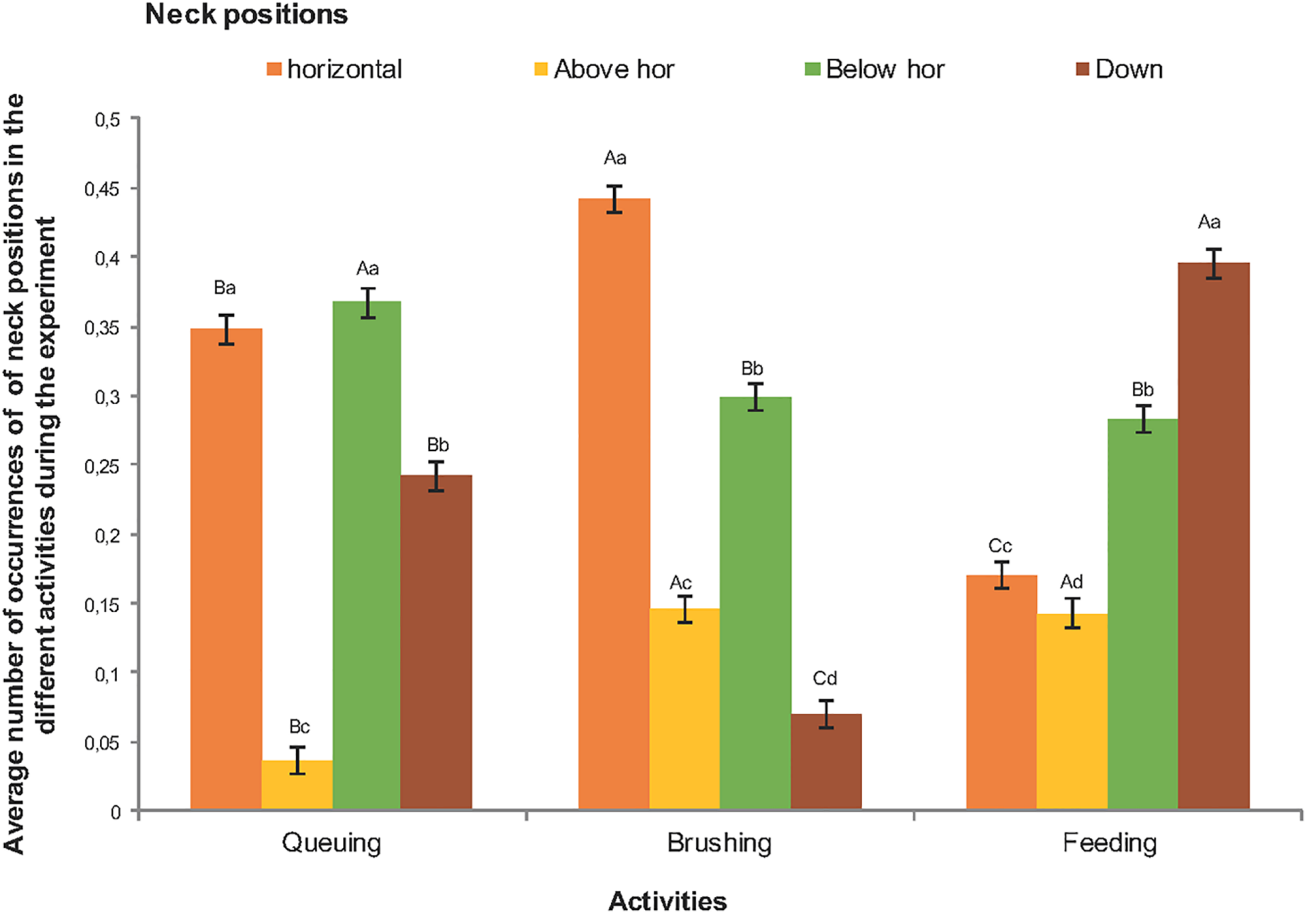
Average number of occurrences (±SE) of horizontal, above horizontal, below horizontal and down positions by dairy cows while queuing, brushing and feeding in the loose housing stable. Different lower-case letters (a, b, c, d) means that there is a difference within activities whereas different upper-case letters (A, B, C) means a difference between activities. Significant differences are presented at the 0.05 level.

There was a significant interaction between neck position and activity (F = 96.37, df = 6, df error = 15035, P<0.0001, Fig 3). The neck down position was most common while feeding, next most frequent during queuing and least frequent during brushing. Typical of queuing for the AMS was that the neck was below the horizontal more often and above the horizontal less often than for any of the other activities. The horizontal neck position was most frequent during brushing.

### Tail positions

**Fig 4:**
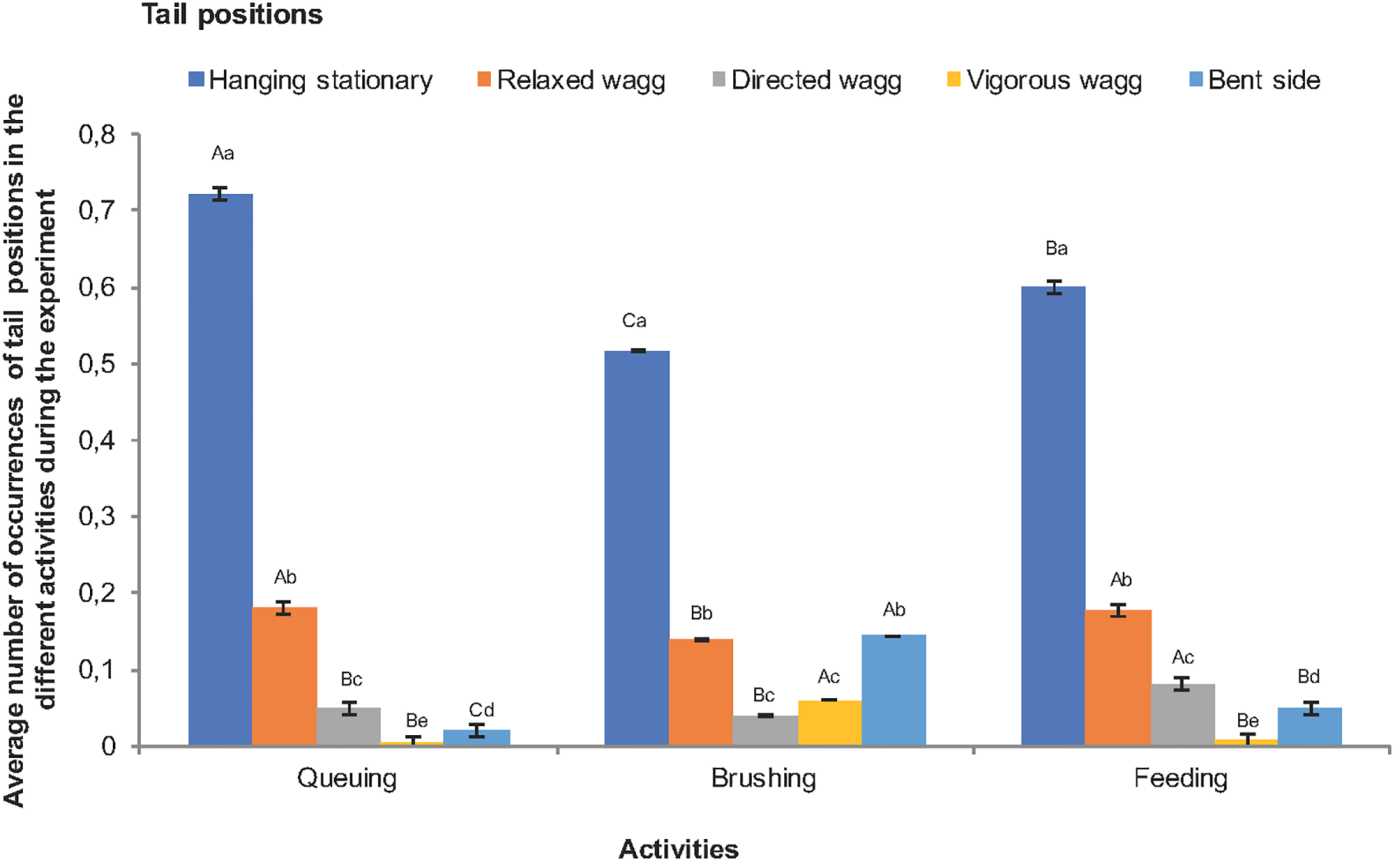
Average number of occurrences (±SE) of no wagging, small wagging, directed wagging, vigorous wagging and the bent side tail position by dairy cows while queuing, brushing and feeding in the loose housing stable. Different lower-case letters (a,b,c,d) means that there is a difference within activities whereas different upper-case letters (A,B,C) means a difference between activities. Significant differences are presented at the 0.05 level.

There was a significant interaction between tail position and activity (F = 34.71, df = 8, df error = 18234, P = <0.0001, Fig 4). Having the tail hanging stationary downwards was the most frequent position in all activities, although it was more common during queuing and least common during brushing. Vigorous tail wagging and the tail being bent to the side were most common during brushing, whereas tail wagging directed at the body was most common when the cow was feeding.

### Principal component analyses

The overall PCA analysis of all the different ear, neck and tail positions shown during the three activities accounted for 65.8% of the variance of the data and resulted in 6 orthogonal rotated factors with an eigenvalue higher than 1 (Table 1).

**Table 1:** Loadings of the first six components extracted by principal component analysis (PCA), after varimax rotation of cow´s ears, tail and neck positions recorded while queuing, brushing and feeding.

Variable	Body part	PC1	PC2	PC3	PC4	PC5	PC6
**Above horizontal**	Neck	**0.59**	0.03	-0.15	0.07	-0.12	-0.04
**Back up**	Ears	**0.46**	-0.04	0.08	-0.17	-0.03	0.17
**Back down**	Ears	**0.42**	0.15	0.20	0.17	-0.03	**-0.48**
**Horizontal**	Neck	-0.12	**0.51**	-0.02	-0.22	-0.25	0.04
**Down**	Neck	-0.07	**-0.73**	-0.02	-0.03	-0.15	-0.05
**Hanging stationary**	Tail	-0.02	-0.04	**-0.66**	-0.09	0.18	-0.10
**Small wagging**	Tail	-0.002	-0.01	**0.52**	-0.14	0.15	-0.23
**Axial**	Ears	-0.03	-0.18	-0.02	**0.66**	0.03	0.03
**Directed wagging**	Tail	-0.1	-0.25	0.34	**-0.41**	0.05	0.15
**Below horizontal**	Neck	-0.20	0.23	0.15	0.28	**0.59**	0.02
**Bent side**	Tail	-0.15	0.03	0.24	0.39	**-0.40**	0.19
**Asymmetric right**	Ears	0.24	0.07	0.08	-0.03	0.22	**0.46**
**Asymmetric left**	Ears	0.05	0.06	-0.02	0.07	-0.09	**0.62**
**Vigorous wagging**	Tail	0.13	0.04	0.09	0.11	-0.36	-0.001
**Forward**	Ears	-0.32	0.16	-0.08	-0.05	-0.37	-0.14
**Variation**		17.0%	15.3%	9.8%	9.3%	7.5%	7.0%

The complexity of the data was shown by the number of extracted components and that in the majority of them, only two out of the 15 variables loaded higher than 0.4 in each component. The exception was PC1, which loaded 3 variables; neck position above the horizontal and ears being either back-up or back-down. Noteworthy is that ear position back-down is the only position that loaded above our threshold on two different components. The other two most frequently occurring neck positions (horizontal and down) loaded on component 2, and the two most frequently observed tail positions (hanging stationary and small wagging) loaded on component 3. Equally important, this overall PCA shows which ears, neck and tail positions are clearly unrelated to each other, by the fact that they loaded on different components.

**Fig 5:**
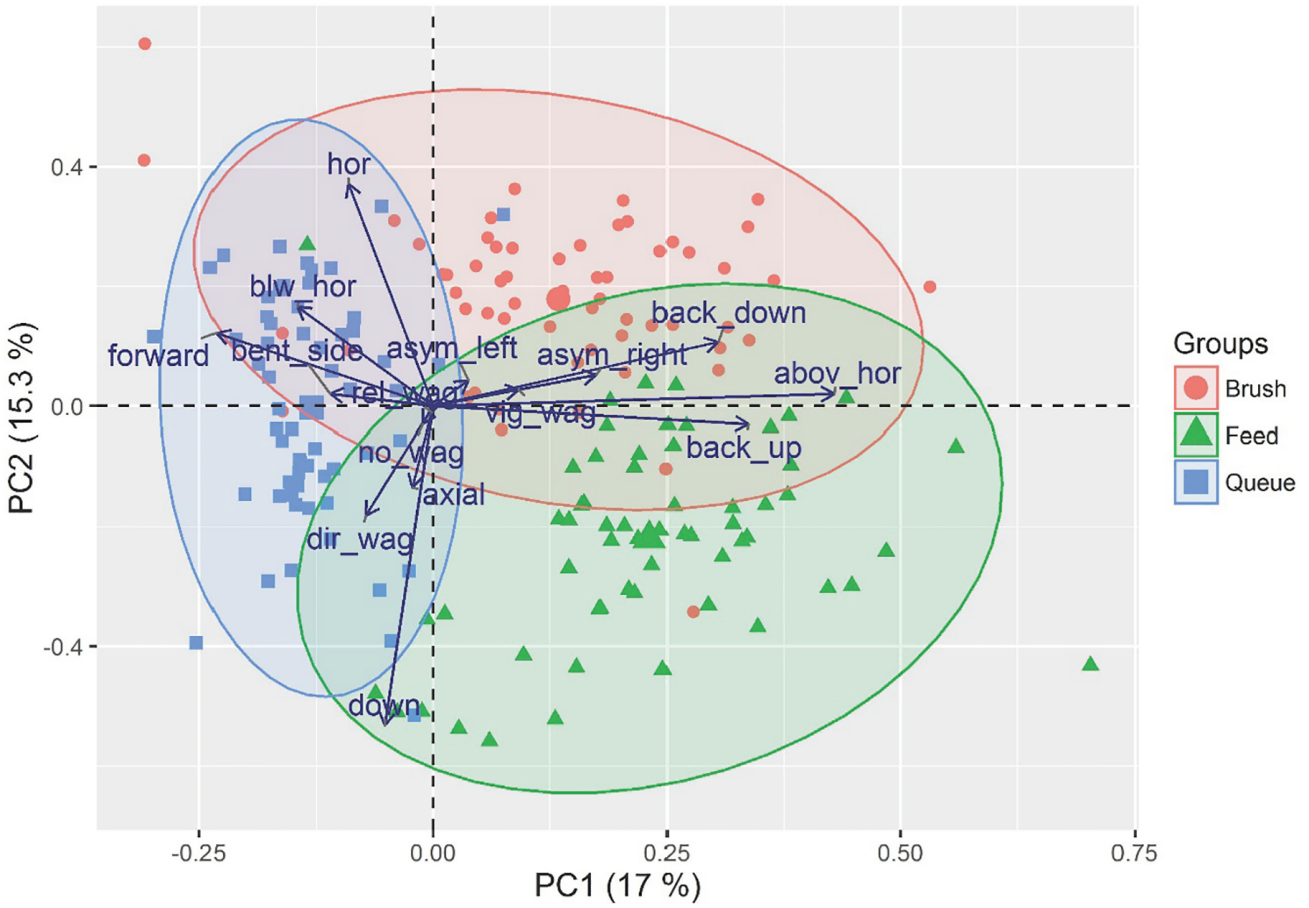
Biplot of the PC1 and PC2 loadings showing cow’s body postures observed during brushing, feeding and queuing in a loose house system and the 95% cluster for each of these three behavioural activities.

To better illustrate how different ear, neck and tail positions clustered among the different activities, we plotted the data in concentration ellipses in component 1 and 2 (Fig 5). The different activities clustered specific body positions and a clear separation was shown between the three activities. Queuing (blue) clustered negatively on component 1, whereas both brushing (red) and feeding (green) clustered positively on component 1. However, these two activities could still be distinguished from one another because brushing clustered positively on component 2 whereas feeding clustered more negatively on this component.

## Discussion

Our results show that by their body postures, cows express themselves differently when performing different activities in the barn, even if these activities all occur when the animals are standing still. We discuss these findings in the light of observations of body postures by other researchers. The main part of this discussion section, however, relates to how this study can be used to investigate which body postures might be related to the different dimensions of emotion. For this more fundamental question we use our results to generate predictions related to the valence and arousal characteristic of specific ear, neck and tail positions, that we propose could be tested under more controlled experimental situations to validate their usefulness as reliable indicators of emotion in cattle.

In this study, we selected three focus activities where cows were stationary and recorded body positions that were potentially unrelated to the performance of the behavioural activity itself. Even though some overlap of the 95% clusters for the behavioural activities of feeding, queuing and brushing was present in the PCA plot, there is a relatively clear separation between these three activities across the two components. The queuing activity cluster is spread along the second component and negatively clustered on component 1, whereas brushing and feeding are positively loaded on this component, but are loaded oppositely on component 2. This illustration, in combination with the statistical analyses of the ear, neck and tail positions, makes it possible to describe the unique body postures associated with feeding, queuing and brushing. The body posture of a feeding cow was ears back up, neck down and directed tail movements. For a cow that was queuing to gain access to the AMS it was ears axial and forward, neck below the horizontal and the tail hanging stationary. For a cow that was brushing it was left ear backwards, neck horizontal and vigorous tail wagging.

An interesting finding in our study relates to the ear positions back up and back down, which were loaded positively on PC1 and both were significantly more frequent during brushing and feeding than during queuing. These ear positions have often been reported previously. In sheep and goats, the position ears backwards was associated with unpleasant and negative emotions [10, 13], whereas in other studies with sheep and cattle it has been associated with positive states [11, 15, 27]. Of particular interest is the position ears back down. This ear position is similar to the ear backwards described in [15]. The authors concluded that this ear position together with ears perpendicular (similar to our axial) reflects a low arousal and positive emotional state. In our study, ears back down was the only body position of the 15 included in this study that loaded highly on two different components. This implies that it may have low specificity, being used in different contexts by animals, a view which supports earlier discussions of ear positions (see commentary by Ekman on page 64 in [7]).

This study was the first to assess systematically a variety of different body postures in cattle from a holistic point of view and the novelty of this analysis brings new knowledge to the understanding of expression of emotion in cattle. Specifically, it might help to pin point specific postures to be investigated further and that can be key to understanding how cows are experiencing different situations. For instance, asymmetric ear positions were assessed for the first time in cattle in this study and were more frequent during brushing suggesting that further studies of such asymmetries in cattle may be justified. Previously in sheep (in experimental set ups) an asymmetric ear posture was associated with sudden and negative stimuli [10, 11], but it has also been shown in positive situations such as play [28].

Neck postures have only been poorly investigated and, to our knowledge, tail postures in cattle have never been studied during routine activities before. Our results have shown that the two most frequently occurring neck positions (horizontal and down) accounted for a greater proportion of the total variation in the data than the two most commonly occurring tail positions (hanging stationary and small wagging). This highlights the importance of investigating neck position, as well as including it when studying overall body posture. So far the only reference in the literature is to stretching the neck, which was associated to positive emotions [29, 30]. Another interesting finding relates to vigorous tail wagging in cows. This posture almost only occurred during brushing and loaded positively in PC1. Previous research describing tail movements in cattle are those investigating the consequences of tail docking [31, 32]. However, in other species, tail wagging and postures have been investigated and associated with different emotional states (dogs [33], sheep [11], goats [13] and pigs [14]). This might be the case for cattle as well.

Emotion is often described in relation to two characteristics, their valence (how positive or negative it is) and the level of arousal that is involved [24] [25, 26]. In the following section, we speculate on how the results from this study can contribute to our knowledge on how body posture is associated with emotional valence and arousal in cattle. We do this without attributing any discrete emotional states to cattle or speculating on the conscious component of emotion. For a discussion on integrating discrete and dimension approaches of emotion see Mendl et al. [26].

**Fig 6:**
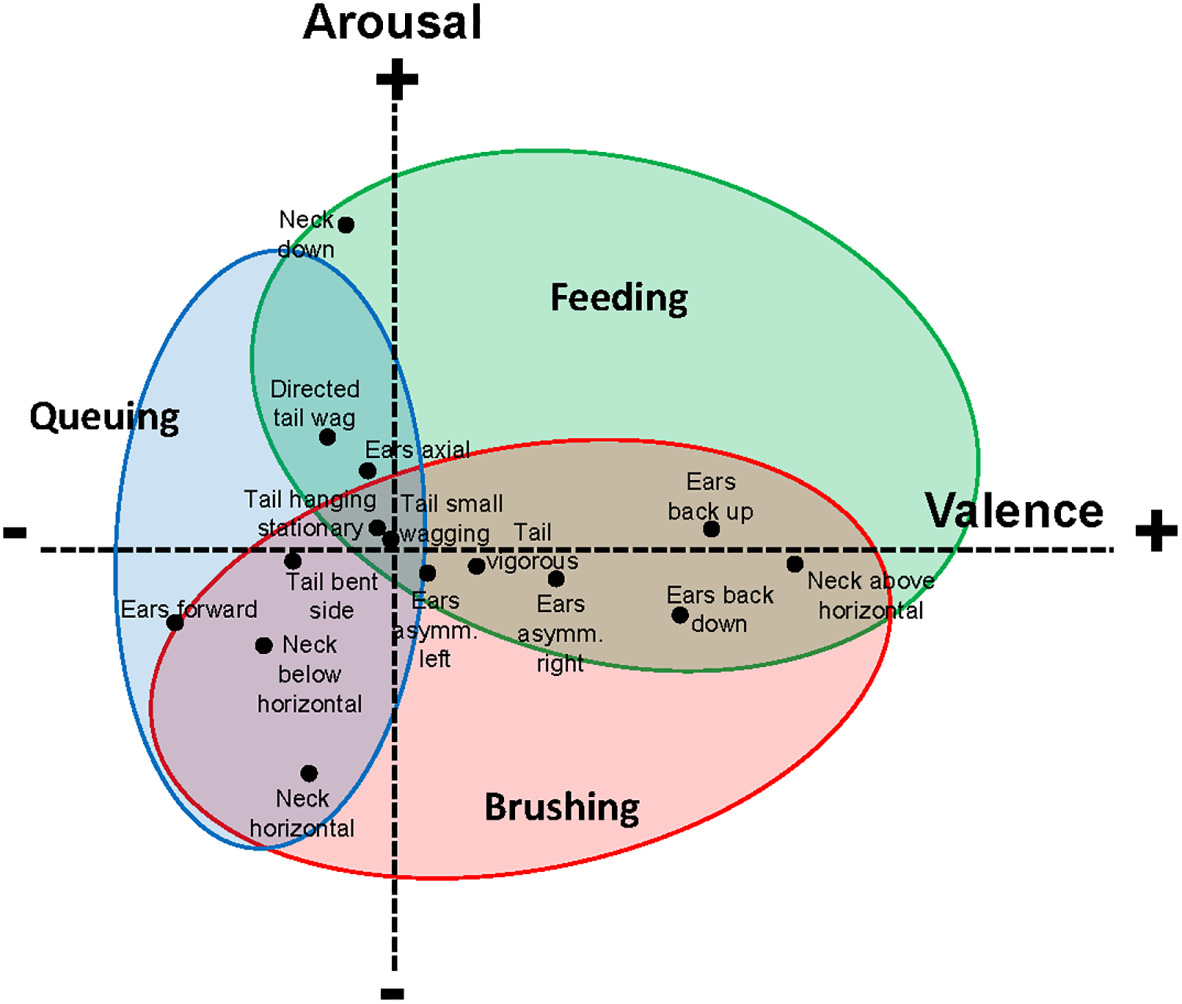
Hypothetical representation of the core affect diagram in two-dimensional space, illustrating ear, neck and tail postures of dairy cows during brushing, feeding and queuing in a loose housing system.

In the stylised Fig 6 we have kept the clusters around the activities of feeding, brushing and queuing in the same position relative to each other. We have named the first component ‘valence’ since there is strong evidence to suggest that brushing and feeding are experienced positively by cattle [15, 29, 30, 34, 35]. We have named the second component ‘arousal’, but have rotated the PCA figure along its horizontal axis since there is work in cattle to support that being brushed is associated with lower heart rate (HR) [36, 37]. This puts low arousal on the lower part of the graph, which is the direction in which it has been represented previously [24, 26]. Having orientated our figure in accordance with existing research on dairy cattle and the dimensional framework for research on emotion, we now use this to generate predictions.

A first major issue, however, is the question of how appropriate it is to name the first two components ‘valence’ and ‘arousal’. We suggest that this question itself leads us to generate new predictions. With regard to the naming of the valence axis, as stated previously we know that animals approach feed and approach to be brushed [35, 38], implying that this is experienced as positive, but we are not aware of work on the emotional valence associated with queuing. Its position relative to feeding and brushing in the analysis of our data leads us to suggest that it is mainly experienced negatively by cows. This prediction could be tested. One could speculate that although positive anticipation of being milked and getting the small feed reward [39] may lead the cow to the waiting area, the long waiting time before she actually enters the AMS may lead to queuing being experienced more negatively, since it has been shown to reduce the time spent eating and resting [40]. Cows in this study were spending approximately 10% of their daytime queuing to be milked and it has been shown that low ranked cows stay in the queue for 35% longer time than high ranked cows [41, 42]. With regard to the naming of the arousal axis, then we would predict from Fig 6 that HR (an indicator of arousal) would be very variable during queuing as this cluster spreads over a wide range on this axis. Following on from our previous speculations about queuing, we might predict that HR would be high when they first enter or are about to leave the queuing area, reflecting positive anticipation, but low in the middle, reflecting a period of inactivity. This could be tested in the future since HR recording is well developed in cattle [43].

Our knowledge of feeding and brushing was used to orientate the mapping of these activities in affective space, but there were no *a priori* assumptions about the valence or arousal associated with queuing. As important, is neither were there any *a priori* assumptions about what a specific body posture tell us about the valence or arousal levels of the cow with that posture. Fig 6 helps us generate predictions that can be tested by observing whether these specific body postures are seen in other activities with known levels of arousal or valence. For example, is neck above the horizontal associated with other positively valenced situations? The work with positive anticipation in poultry [44] where the neck is stretched upwards, would suggest that it does. This approach also leads to speculation about the biological relevance of specific body postures connected to emotion and the extent to which they are adaptive for the animal [4, 7, 45].

From Fig 6, we would also predict that having the right ear back (ears asymmetrically right) is associated with more positively valenced emotion than having the left ear back. The right side of the body is controlled by the left side of the brain, so this prediction is indeed in keeping with the currently accepted view of a lateralization pattern for emotional processing, where there is a left-hemisphere dominance for processing positively connotated emotions, such as those elicited by a food reward [46]. There is also support for the corresponding prediction, that having the left ear back (asymmetrically left) would be associated with negatively connotated emotions. During social interactions, losing and subordinate cows were more likely use the left eye to look at winning, dominant cattle and unfamiliar humans [47]. A left ear asymmetry in cows in negative situations is also in keeping with studies on different species, such as sheep, horses, cats and rats, showing that stressful situations activate the right hemisphere of the brain [48].

Ears forward loaded on the negative side of the valence axis, which could lead to the prediction of an association between this ear position and more negative emotion. In support of this prediction, previous literature associated ears forward with negative situations in sheep [10, 11] and this ear position has been shown by reactive/anxious rats when approaching a novel stimulus [49]. As stated earlier, previous research on ear position seems to be contradictory and it may be that a possible explanation for the apparent inconsistency is that the animals in those studies were at different levels of arousal rather than necessarily in states with different emotional valence. We have mentioned previously the position where both ears are pointing backwards, which has been associated to both positively and negatively valenced situations [16]. A suggestion, as a prediction to be tested from our study, is that ears back up would be associated with a more highly aroused emotional state than ears back down.

Regarding tail wagging there is a clear gap in our knowledge relating it to emotional expression in cattle. Nevertheless, we can speculate, based in our results, that tail wagging in cattle might be associated with positive valenced emotion. This is based on the stronger loading of vigorous wagging on the valence axis than tail hanging stationary, but their rather similar loadings on the arousal axis. Other previous research in different species could support this prediction, since moving the tail generally occurs in positive situations [13, 14, 33].

The approach used in this study allows us to formulate specific predictions associating body postures with emotion. Nevertheless, we cannot ignore that some specific body postures might be associated with the mechanics of the behaviour itself. For instance, the position neck down while feeding may be influenced by the height of the roughage bin, or how much food is in it, and the position neck above the horizontal when brushing may be influenced by the height of the mechanical brush during the time that the cow is actively manipulating it. Future research testing the predictions generated in this study should pay special attention to these potential confounders.

A further issue is to what extent we have managed to cover the full range of positive to negative valence and high to low arousal in our cows. All cows were in apparent good heath, with no obvious indications of pain during the observation period. On the other hand, being in a high producing milking herd under commercial housing conditions, even if well managed, is presumably rarely associated with a very positive emotional state [50, 51]. Similarly, with few exceptions, the cows probably did not experience the extremes of arousal during our observation periods [43, 52]. We therefore suggest that our body postures represent a relatively limited range of the valence and arousal variation than could potentially be generated under experimental conditions or that might occur in animals in the wild. Neither do we know if we are correct to talk about positive and negative valence *per se*, since we have no knowledge about where our cows are in relation to the possibly ‘neutral’ experience at the point of origin of these dimensions. For these reasons we have been careful to make predictions referring to body positions as reflecting a more or less positive, or more or less negative valence. However, in the probable absence of more extreme primary negative emotional states like pain and fear, or the presumed positive ones like play, this dimensional approach might be the most appropriate method to investigate emotion in animals in their normal daily life. By using it we can avoid arbitrary assumptions, for example by associating body postures with discrete emotional states, while still being able to provide insight into emotion by formulating specific predictions related to the valence and arousal of the emotion associated with a body posture that can be tested and validated in the future.

Further limitations include that it was not possible to have this as a blinded study, since cows were selected according to the activity they were performing. One can also discuss if our observers were sufficiently well trained, or if the results would have been different in another design of loose housing or with another herd. These are valid concerns if a study is to be replicated. However to be useful in practice, indicators of emotional valence and arousal should be widely applicable for different people and for cows in different situations. The key test of the worthiness of the methodological approach proposed here is whether the *a priori* predictions are supported by new, independent studies in the future. Particularly useful would be if they could be tested in situations where the emotional valence and arousal are systematically changed and where corresponding observations in body posture are combined with physiological measures. The usefulness of this new methodological approach therefore remains to be confirmed and the results here should be considered as a first step. Our long term aim is to contribute to the development of reliable indicators of cow emotion that could in the future be used in welfare assessment protocols, as well as contribute to a methodology that can be used to link body posture to emotional valence and arousal in other species.

In conclusion, our study has shown that cows express different ear, neck and tail positions in the barn, and we were able to use this behavioural variation to compose the typical body postures associated with different activities.
